# A novel digital approach for fixed full-mouth implant-supported rehabilitations: A case report

**DOI:** 10.4317/jced.57208

**Published:** 2020-09-01

**Authors:** Arturo Llobell, Michael Bergler, Howard Fraiman, Jonathan Korostoff, Caleb Cross, Joseph Fiorellini

**Affiliations:** 1 Periodontal Prosthesis Graduate Program, Periodontics Department, University of Pennsylvania

## Abstract

Successful rehabilitation of a patient’s entire dentition with implant-supported fixed prostheses requires restoration of function, esthetics and comfort. To achieve this goal, the clinician and laboratory technician must work in concert with one another to navigate the multiple steps from the patient’s initial evaluation to delivery of the final prostheses. Key to this is the ability of the clinician to provide the technician with detailed information regarding the patient’s extra- and intraoral characteristics in a manner that can be easily and accurately transferred to the lab bench where it then serves as the foundation for reconstruction of the dentition. In recent years, the impressive evolution of digital technology in dentistry has dramatically facilitated this complex process. The aim of this case report is to illustrate how digital profiles of a patient’s facial and intraoral features can be merged with one another and used to generate artificial teeth and gingival tissue of a full mouth implant supported rehabilitation via computer-aided design and computer-aided manufacturing (CAD/CAM) technology to successfully rehabilitate a patient that initially presented with a terminal dentition.

** Key words:**Facial scan, Zirconia, Implant-supported rehabilitation, Implant-supported prosthesis, Fixed prosthesis, Oral rehabilitation.

## Introduction

The multidisciplinary approach to dental rehabilitation of partially or fully edentulous patients has evolved significantly in recent years (1-4). The changes have been driven by clinicians’ desire to achieve more predictable, longer lasting outcomes without compromising the patient’s comfort, esthetics and function. The evolution has manifested itself in both the surgical and prosthodontic aspects of interdisciplinary treatment, amongst others, through the development of new materials, techniques, fabrication processes and digital design capacity.

This case report describes a novel approach in digital dental technology as it is the first to utilize merged three-dimensional (3D) extra- and intraoral scans of a fully edentulous patient. The merged scans were then used to digitally design maxillary and mandibular fixed implant-supported prostheses to restore both the patient’s teeth and the associated gingival tissues. The application of this novel facial scanning and digital design technology resulted in successful rehabilitation of the patient satisfying both his functional and esthetic demands.

## Case Report

A healthy male patient with a terminal dentition presented to our office for a full-mouth rehabilitation. At the first visit, the patient expressed his concerns and desire to restore both the esthetic and functional aspects of his dentition with fixed prostheses. The patient had undergone previous dental treatment that included tooth-supported full contour fixed partial restorations and extractions.

**Figure 1:**
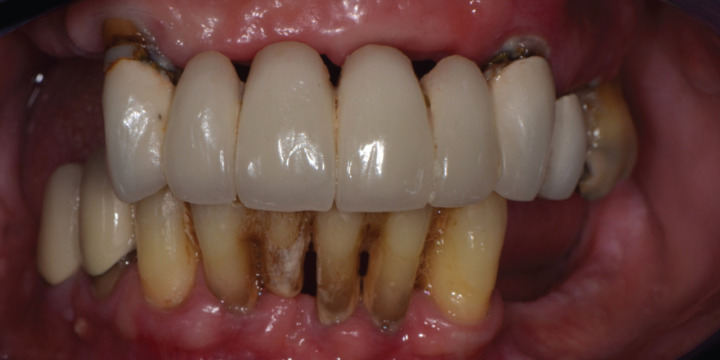
Initial situation.

Clinical examination (Fig. 1) revealed partial edentulism, a significant number of carious lesions, deep periodontal probing depths, generalized gingival recession and tooth mobility. The maxillary arch exhibited a metal-ceramic fixed partial denture that spanned from tooth number 13 (class 3 mobility) to 24. Due to recurrent caries on a majority of the abutment teeth, the prosthesis was supported mainly by number 13. The mandibular arch exhibited partial edentulism, metal-ceramic crown restorations on the right premolars, the crown of a previously extracted incisor bonded to the adjacent teeth, deep periodontal probing depths, recession and a lack of keratinized tissue in the posterior sextants. The clinical findings were consistent with a diagnosis of severe chronic periodontitis affecting the remaining dentition.

A panoramic radiograph demonstrated advanced bone loss in the posterior sextants and confirmed the presumptive clinical diagnosis of severe chronic periodontitis. A cone beam-computed tomography (CBCT) scan revealed generalized atrophy of the maxillary alveolar process, bilateral sinus pneumatization and a horizontal pattern of bone resorption in the premolar area. Two impacted teeth communicating with the nasal cavity and a horizontal pattern of bone resorption were detected in the anterior maxilla. In the posterior sextants of the mandible the scan showed a vertical pattern of bone resorption with the ridge crest in close proximity to the inferior alveolar nerve.

A final multidisciplinary treatment plan was presented to the patient that included numerous extractions, guided bone regeneration, bilateral sinus augmentation procedures, mucogingival procedures, implant placement and ultimately, insertion of full-arch maxillary and mandibular implant-supported fixed prostheses. The patient was informed that the prostheses would be designed utilizing the novel approach of merging his facial scan with a verified digital file of the existing intra-oral condition.

-Treatment Sequence

Following atraumatic extraction of the remaining teeth, alveolar ridge preservation procedures using freeze-dried bone allograft (3i) and resorbable collagen membranes (Geistlich Bio-Guide) were performed. Immediate removable full dentures were inserted to restore the patient’s occlusal function and facial esthetics. Prior to commencing with additional surgery, a healing period of six weeks took place allowing for soft tissue healing, adequate relining of the immediate dentures and patient habituation.

A. Surgical Treatment 

Maxillary Arch

Full thickness flaps where elevated in order to remove the two impacted teeth in from the anterior maxilla. Following extraction removal, the nasal floor was sealed with a resorbable collagen membrane (Geistlich Bio-Guide). The surrounding cortical bone was perforated and a combination of xenograft (Bio-Oss) and recombinant human platelet-derived growth factor-PDGF; (Gem-21 Lynch Biologics) was placed in order to regenerate the bone of the anterior maxilla. A well-adapted titanium mesh was used to retain the graft in the desired position. Prior to closure, the mesh was covered with a resorbable collagen membrane previously soaked in rh-PDGF. Ten months after the extractions and bone regeneration procedure, the site was re-entered revealing successful vertical and horizontal bone augmentation. Implant placement was subsequently performed (Zimmer- Biomet 3i).

In the premolar area, a ridge split crest technique followed by simultaneous implant placement was performed. A xenograft (Geistlich-Bio-Oss) was placed around the implants and the site was covered with a resorbable collagen membranes (Geistlich-BioGuide).

Simultaneous bilateral sinus augmentation procedures with implant placement were performed in the posterior maxilla. The Scheniderian membrane was elevated without perforation or complication. Xenograft (Geistlich Bio-Oss) was placed in each sinus and osteotomies were prepared for implants. Implants (Zimmer-Biomet 3i) were inserted without complications and all of them exhibited sufficient primary stability as measured by the insertion torque. A resorbable collagen membrane was placed over each sinus window prior to flap closure.

After 4 months, the implants were exposed via elevation of full thickness flaps. The incisions were designed with the intention of preserving as much keratinized tissue as possible. The cover screws were removed and replaced with Low Profile abutments (Zimmer-Biomet3i). The flaps were then sutured in place around the abutments.

-Mandibular Arch

The mandibular implants were placed using a guided approach. Computer guided software (Simplant) was used to design the guide with the intention of placing the implants in positions that allow for an ideal functional and esthetic prosthetic outcome. Due to the limited amount of keratinized tissue, a bone supported guide is preferred. A mid-crestal incision was made along the entire mandibular arch. Full-thickness buccal and lingual flaps were elevated and the guide was stabilized by seating it on the alveolar process. A total of four implants (AstraTech) were placed between the mental foramena. Two short implants were inserted in each posterior segment due to the compromised alveolar bone height.

During the implant osseointegration period, free gingival grafts from palatal donor sites were done in order to augment the keratinized tissue around the mandibular implants.

After allowing 4 months for successful implant osseointegration and adequate healing of the soft tissue grafts, the implants were exposed via elevation of full thickness flaps. The cover screws were removed and replaced with Uni-Abutments (AstraTech). The flaps were coapted in a manner that allowed for preservation of keratinized tissue.

B. Prosthodontic Treatment

Following exposure of the implants, the immediate dentures were converted into fixed implant-supported provisional prostheses through the luting of temporary cylinders connected to the Low Profile and Uni-Abutment final abutments. This was done in a manner that maintained the occlusal and inter-arch relationships that where previously established,

Conventional open-tray implant impressions were made with a vinyl polysiloxane material (Heraeus-Kulzer) that were then utilized to fabricate the respective master models. Metal-reinforced verification jigs were fabricated and placed intraorally to confirm the accuracy and precision of the master models by evaluating the passivity of fit of the jigs.

The verified master models were transferred to a semi-adjustable articulator (Artex Amman-Girrbach) with the use of the existing fixed provisional restorations. The verified and articulated master models were then scanned using a laboratory extra-oral scanner (Zirkonzahn) and merged with a series of facial scans of the patient. Frontal and side view scans were made with the patient in rest, smile and retracted cheek positions using a Face Hunter scanning device (Zirkonzahn).

**Figure 2:**
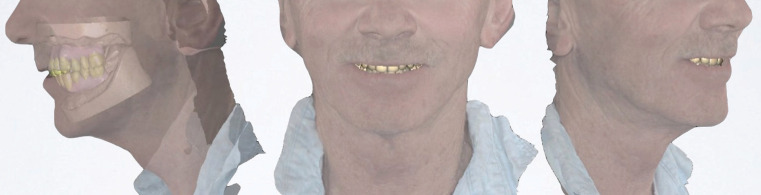
Merging of a section of the full-face facial scan with the CAD design of the implant-supported prosthesis.

Avoiding the use of a new face bow registration, conventional articulator, inter-arch relationship registrations and multiple try-ins, the final prostheses were designed and fabricated digitally after merging the facial scans and master model digital files (Fig. 2).

This design was first milled in PMMA (Zirkonzahn) in order to clinically verify the accuracy of the prostheses and ensure adequate function as evaluated by the patient’s speech and mastication. The patient tested the provisional versions of the prostheses for six months.

At the six-month re-evaluation, impressions of the provisional restorations were taken in order to fabricate models that were scanned and superimposed with the original design. This extra step was taken in order to: 1) identify possible occlusal wear facets that developed during the six-month evaluation period and 2) finalize the design of the final prostheses.

**Figure 3:**
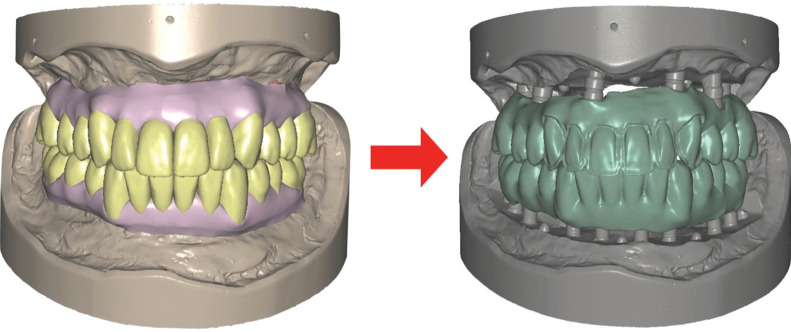
CAD Design of the PMMA and final prosthesis.

Upper and lower final prostheses where fabricated following a monolithic zirconia design, with the exception of teeth 13-23 that had facial/buccal cutbacks in order to layer porcelain for ideal final esthetics (Fig. 3).

**Figure 4:**
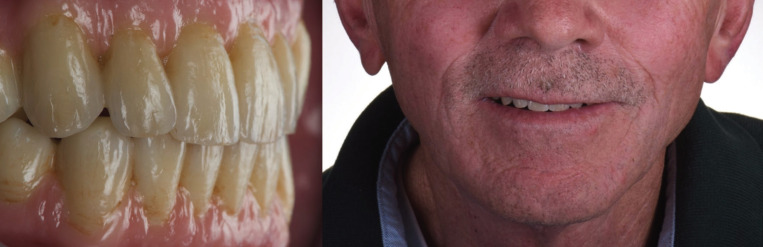
Clinical outcome at 4-year follow-up since delivery of the final prosthesis.

Upon delivery of the final upper and lower prostheses, the initial goals of restoring comfort, function and esthetics where achieved. The patient was pleased with the tooth shape, size, color and smile arc definition. At follow-up visits the patient denied any issues relating to the function and comfort of the prostheses. Furthermore, the patient was free of prosthetic and implant complications at his re-evaluation visits 6.5 years after implant placement and 4 years after final prosthesis were delivered (Fig. 4).

## Discussion

The facial scan has been previously used in dentistry (4-8), although this use has been restricted to surgical planning, cone beam computed tomography (CBCT) evaluation, orthodontic therapy and/or fixed partial prosthesis. Currently and up to the knowledge of the authors, no article has been published regarding full mouth implant supported rehabilitations using fixed implant supported-dentogingival prosthesis and according to the date when it was performed, assumption can be made that this was the first dentogingival implant-supported rehabilitation that followed this protocol. This case report illustrates the advantage of planning both full arch prosthesis with the use of a face scanner, avoiding multiple try-ins and knowing exactly where the esthetics lay during the prosthetic design and prior to the fabrication of any prosthetic structure or try-in.

The use of the facial scan made the prosthetic design easier for the lab technician, relating every change in tooth macrogeometry to the shape and size of the patient’s face and smile. The designed followed this novel protocol and was then carried up to the fabrication of the final prosthesis, achieving successful functional and aesthetic outcomes. Although proving beneficial in this specific case, the use of a facial scan in prosthodontics must be implemented with caution as there is a lack of sufficient scientific evidence of it´s precision due to the novelty of the approach and further research in these aspects is therefore warranted.

The use of zirconia as the material of choice for the final prosthesis proved beneficial in terms of mechanical properties (9-10) improved esthetics, adequate fit (11) and low bacterial adhesion (12). This was however achieved along with potential limitations including possible long-term hydrothermal degradation (13) and it’s difficulty to adjust/repair prostheses after sinterization (14).

The porcelain veneer cohesive factor has been frequently debated in recent years due to the high rates of porcelain veneer chipping over zirconia frameworks (15-17). These fractures have been mainly related to insufficient support of the veneering material by the framework design (18), mismatch of coefficients of thermal expansions between porcelain and zirconia, unfavorable zirconia surface treatment and possible ceramic strength degradation over time. Despite the significant number of articles describing the fracture of this porcelain layer over a zirconia substructure, results from a recent study concluded that all veneering ceramic/zirconia combinations showed significantly higher bond strength than the metal-ceramic control group (19). The clinical scenario illustrated in this article included a porcelain veneer on the facial aspect of the maxillary anterior teeth to improve the esthetic outcome and did not present complications upon follow-up evaluations (3,20-22).

Two different types of dental implants have been placed in the present case report. The decision to use different implant systems was based on our evaluation of bone morphology and quality in each arch. In the upper arch where implants where placed in either grafted bone or simultaneous with bone augmentation procedures, a tapered body design implant was utilized in order to achieve higher insertion torque, a measurement that has been correlated with higher implant stability (23,24). In the posterior sextants of the mandibular arch, a short implant design with an internal connection was chosen (Astra-Tech) due to the limitation in alveolar bone height. Short implants have been described as a successful alternative option to bone augmentation techniques and nerve transposition procedures in this area (25), avoiding the associated increased postoperative morbidity, higher treatment costs, and higher risks of complications during patient rehabilitation (26,27).

The use of autogenous bone as a graft material is considered the gold standard in bone augmentation procedures due to its osteoconductive, osteoinductive and osteogenic properties (28). However, its use usually requires a second surgical site in order to obtain the volume of bone graft needed, increasing the time of the surgical procedure, amount of blood loss, post-surgical morbidity, patient discomfort and surgical cost (29). In the present case, the main choice for a bone graft was a xenograft (Bio-Oss), taking advantage of its slow resorption rate and capacity to maintain bone volume until new bone formation has taken place (30). The osteoinductive nature of the graft was enhanced by combining it with recombinant human platelet derived growth factor-PDGF (Gem-21) (31-34).

## Conclusions

This case report demonstrates that the combination of a facial scan and a digital file of a master model can be combined to achieve a successful outcome in implant dentistry involving complex full arch rehabilitations. This success is defined by the restoration of both function and esthetics in the patient while reducing the number of steps and appointments required without compromising the predictability in the fabrication of the final prosthesis, that followed the design established through the use of a facial scan and maintained throughout the prosthetic treatment phase.
